# Long-term Outcomes of Laparoscopic Sleeve Gastrectomy as a Revisional Procedure Following Adjustable Gastric Banding: Variations in Outcomes Based on Indication

**DOI:** 10.1007/s11695-023-06886-8

**Published:** 2023-10-17

**Authors:** Anagi Wickremasinghe, Yit Leang, Yazmin Johari, Prem Chana, Megan Alderuccio, Kalai Shaw, Cheryl Laurie, Peter Nottle, Wendy Brown, Paul Burton

**Affiliations:** 1 Monash University Department of Surgery, Central Clinical School, Monash University, Melbourne, Australia; 2 Oesophago-Gastric and Bariatric Unit, Department of General Surgery, The Alfred Hospital, Melbourne, Australia

**Keywords:** Bariatric outcome, Laparoscopic sleeve gastrectomy, Bariatric revision surgery

## Abstract

**Background:**

Significant controversy exists regarding the indications and outcomes after laparoscopic adjustable gastric banding (LAGB) conversions to laparoscopic sleeve gastrectomy (LSG).

**Aim:**

To comprehensively determine the long-term outcomes of sleeve gastrectomy as a revisional procedure after LAGB across a range of measures and determine predictors of outcomes.

**Methods:**

Six hundred revision LSG (RLSG) and 1200 controls (primary LSG (PLSG)) were included. Patient demographics, complications, follow-up, and patient-completed questionnaires were collected.

**Results:**

RLSG vs controls; females 87% vs 78.8%, age 45 ± 19.4 vs 40.6 ± 10.6 years, *p* = 0.561; baseline weight 119.7 ± 26.2 vs 120.6 ± 26.5 kg *p* = 0.961)_._ Follow-up was 87% vs 89.3%. Weight loss in RLSG at 5 years, 22.9% vs 29.6% TBWL, *p* = 0.001, 10 years: 19.5% vs 27% TBWL, *p* = 0.001. RLSG had more complications (4.8 vs 2.0% RR 2.4, *p* = 0.001), re-admissions (4.3 vs 2.4% RR 1.8, *p* = 0.012), staple line leaks (2.5 vs 0.9%, *p* = 0.003). Eroded bands and baseline weight were independent predictors of complications after RLSG. Long-term re-operation rate was 7.3% for RLSG compared to 3.2% in controls. Severe oesophageal dysmotility predicted poor weight loss. RLSG reported lower quality of life scores (SF-12 physical component scores 75.9 vs 88%, *p* = 0.001), satisfaction (69 vs 93%, *p* = 0.001) and more frequent regurgitation (58% vs 42%, *p* = 0.034).

**Conclusion:**

RLSG provides long-term weight loss, although peri-operative complications are significantly elevated compared to PLSG. Longer-term re-operation rates are elevated compared to PLSG. Four variables predicted worse outcomes: eroded band, multiple prior bands, severe oesophageal dysmotility and elevated baseline weight.

**Graphical Abstract:**

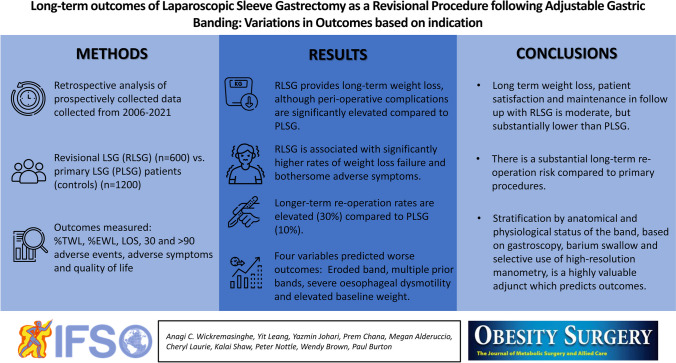

**Supplementary Information:**

The online version contains supplementary material available at 10.1007/s11695-023-06886-8.

## Introduction

Revisional bariatric surgical procedures now represent 10–30% of all procedures performed globally [1–3]. Revisional procedures are technically more challenging than primary procedures; indications are poorly defined, and there are few long-term outcome data [4–6]. Registry data suggest a significantly higher complication rate [3].

Laparoscopic adjustable gastric banding (LAGB) was a widely used bariatric procedure. Long-term studies have shown durable weight loss; however, the long-term follow-up of patients after LAGB has proven to be demanding [7]. Proximal pouch dilatation (40–50%) as well as weight loss failure or regain (10–18%) are frequent causes for intervention or reoperation [7–10]. Laparoscopic sleeve gastrectomy (LSG) is now the most popular bariatric procedure globally [11] and can be used as a conversion surgery following complications of LAGB.

Previous studies reporting on conversion of LAGB to LSG have reported less favourable weight loss in revisional LSG compared to primary LSG, with higher revision rates [12, 13]. The results of these studies were limited by the small number of subjects and have not reported on the patient experience. Whilst complications following LAGB can be objectively classified based on anatomy and specific aspects of oesophageal motility (CORE classification) [14, 15], no previous studies have stratified outcomes of LSG following LAGB based on this objective anatomical or physiological classification of the indication for conversion.

We primarily aimed to report the peri-operative morbidity, long-term weight loss and re-operation risk of LSG performed as a revisional procedure after LAGB. Secondarily, we aimed to report on a broad range of outcome measures. Additionally, as a sub-group analysis, we sought to determine whether indication for surgery based on anatomical and physiological status significantly affected key outcomes.

## Methods

### Study Design

**Fig. 1:**
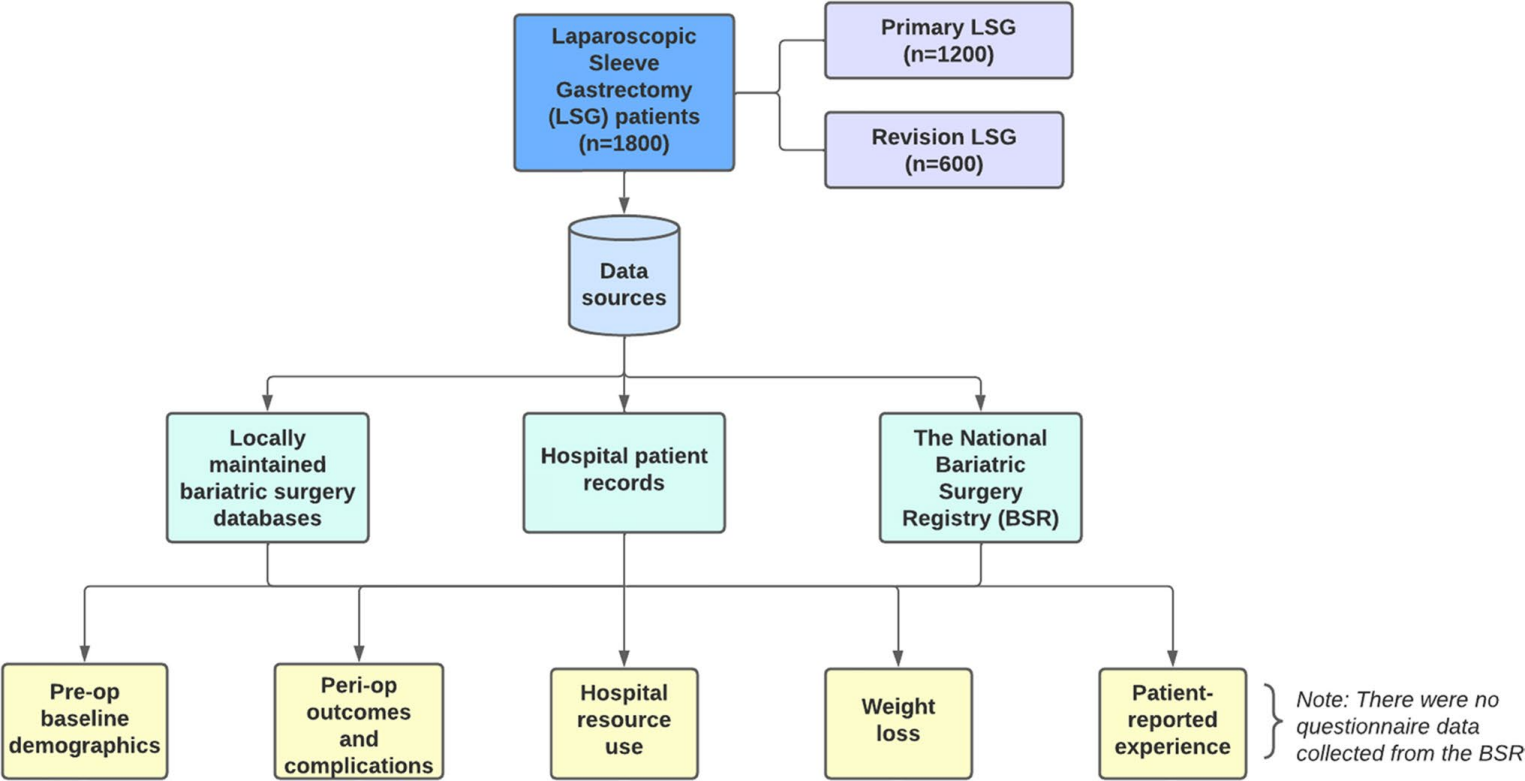
Flow chart of study

We conducted a retrospective analysis of prospectively collected data collected from 2006 to 2021 on the outcomes of LSG patients after LAGB (revisional LSG (RLSG)) comparing outcomes to those of primary LSG (PLSG) patients (controls). Data was collated in a single Microsoft Access™ 2019 database (Microsoft Corporation, Redmond, WA, USA) housed in a university department of surgery. Additionally, data linkages with The National Bariatric Surgery Registry (BSR) were conducted. Figure 1 outlines the study design. A 1:2 analysis of RLSG and controls were performed based on age, gender, pre-operative weight and BMI. Patients were further categorised into indications for RLSG based on the CORE classification for LAGB complications [15].

#### Patient Selection

**Fig. 2:**
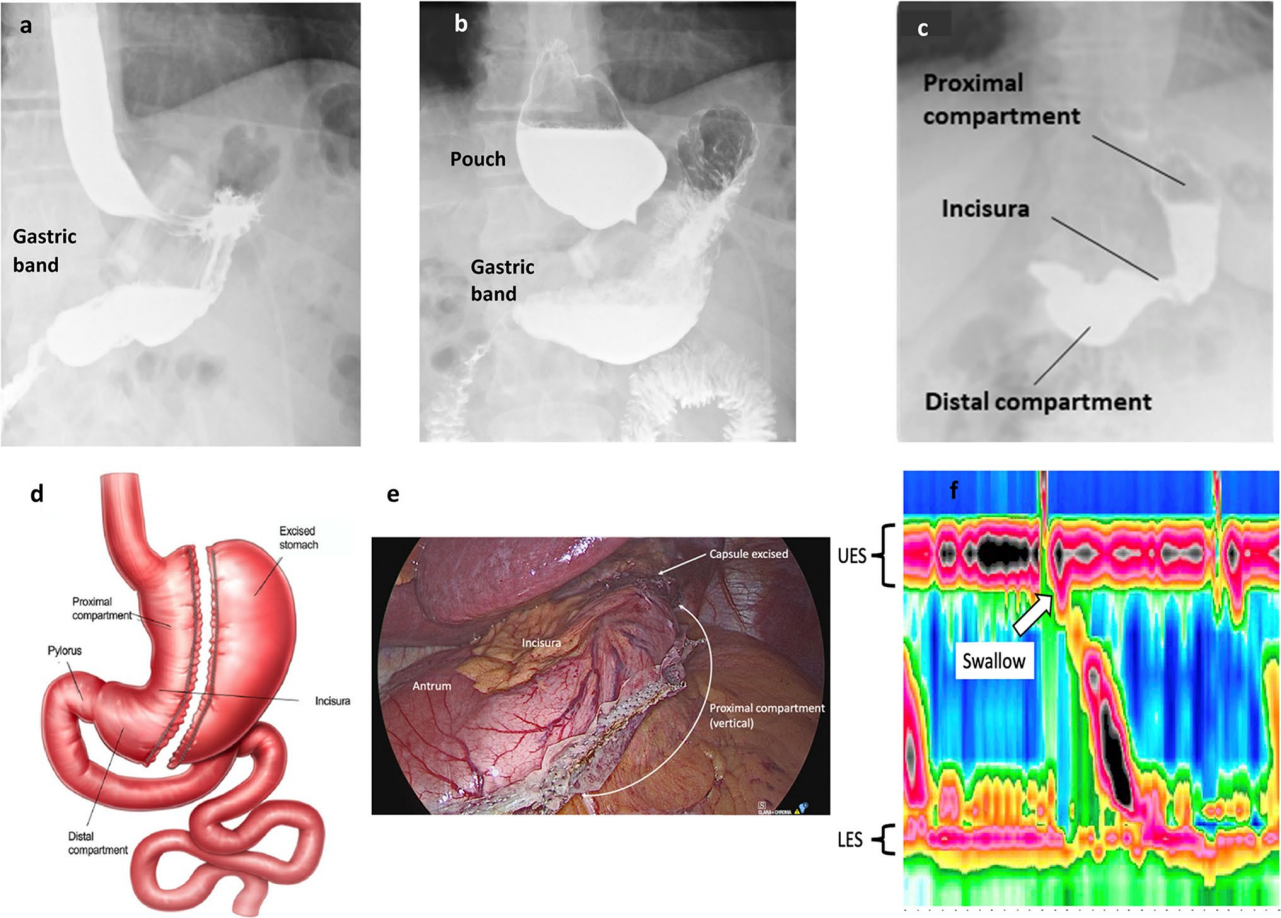
Revisional sleeve gastrectomy. **a** Appearance of a normally positioned LAGB on a barium swallow. **b** Barium swallow images demonstrating pouch dilatations above the band, pooling of contrast above the band with poor emptying of pouch. **c** Appearance of sleeve gastrectomy on a barium swallow. **d** Schematic representation of sleeve gastrectomy. **e** Intraoperative image of a sleeve gastrectomy. **f** Manometric image post revisional sleeve gastrectomy, with impaired lower oesophageal sphincter relaxation

Conversion of LAGB to LSG was performed for patients who had weight regain, adverse symptoms or complications of LAGB. Gastroscopy and liquid contrast barium swallows were performed on all patients seeking conversion of LAGB to LSG to identify proximal pouch dilatation, oesophageal dilatation, prosthetic slippage and erosion into the stomach. High-resolution manometry was performed when there was doubt about the function of the oesophagus [16, 17] (Fig. 2). Any identified complication of the LAGB was classified according to the CORE classification [14].

### Surgical Details

Conversion of LAGB to LSG was undertaken as either a single-stage or two-stage procedure. Single-stage procedures were attempted if gastroscopy, barium swallow and manometry confirmed less than 4 cm of axial separation of the lower oesophageal sphincter (LOS) and LAGB along with four intra-operative factors (Table 1). Any score of III across one of five intra-operative domains or presence of band infection or erosion precluded single-stage conversion. If there were two or more domains scoring greater than II, single-stage was considered contra-indicated. RLSG was performed 3 months after removal of LAGB in two-stage procedures.

**Table 1:** Decision-making algorithm and criteria for single versus two-stage conversion to sleeve gastrectomy: pre-operative and intra-operative factors

	Revision		
	Single-stage (Grade I)	Single/two-stage** (Grade II)	Two-stage* (Grade III)
Preoperative factors			
Band Erosion or infection	Not suitable for single stage		
Axial separation of lower oesophageal sphincter and diaphragm	Not present	< 4 cm	> 4 cm
Stasis within a visible asymmetrical dilated gastric pouch	Not present	Not present	Severe—large volume of liquid semi solid food debris (Fig. 1b)
Intra-operative factors			
Oedema/fluid around band	Minimal	Trace (1–2 ml)	> 2 ml
Quality of gastric wall	Good quality without significant scarring/fibrosis	Moderate thickness with thin layer of capsule amenable to excision	Thin wall with significant attenuation of gastric wall integrity OR excessively thick gastric wall with inseparable and potentially intramural scarring/fibrosis
Shape of gastric wall	No distortion	Minor distortion with dilated proximal gastric compartment	Significant dilatation of proximal gastric compartment forming a large pouch
Surface debris (i.e. embedded suture material)	No visible debris along intended stapling pathway	Visible debris along intended stapling pathway amenable to surgical removal	Excessive debris embedded within intended stapling pathway not amenable to surgical removal
Surface contour	No in folding of gastric wall	Minor in folding of gastric wall from the gastro-gastric tunnel amenable to restoration of normal gastric anatomy	Significant in folding of gastric wall from previous gastro-gastric tunnel not amenable to restoration of normal gastric anatomy

^**^Presence of a trace of fluid was considered to be abnormal

^*^Presence of one of these factors was considered high risk, and a planned two-staged revision was performed

Supplementary Fig. 1 demonstrates intra-operative images of sleeve gastrectomy demonstrating the four intra-operative factors.

### Sleeve Gastrectomy Technique

The technique was similar to that performed in PLSG [18]. For single-stage conversions, following establishment of pneumoperitoneum, the LAGB was removed by dividing the band lateral to the buckle, the gastro-gastric tunnel was dissected, and the stomach fully unfolded. Capsules from the band and gastro-gastric plication sutures were removed to allow for optimal staple line formation. Further adhesiolysis to restore the stomach to the anatomical orientation was performed as required. Once normal anatomy was restored the LSG proceeded.

The stomach was then mobilised along the greater curvature with exposure of the left crus and hiatus, ensuring the posterior capsule was dissected free of the left crus. Small to medium hiatus hernia were identified, reduced and primarily repaired with non-absorbable sutures. A tubularised stomach along the lesser curvature was created over a 36-French bougie by performing a longitudinal resection of fundus, corpus and antrum using laparoscopic linear-cutting staplers. The staple line commenced distally approximately 4 cm from the pylorus and ended proximally about 1 cm lateral to the cardia [18].

The two major types of staplers used were Ethicon Echelon™ Stapler-Echelon Flex™ Stapler with GST reloads (Ethicon Endo-Surgery, Inc., Cincinnati, OH, USA) and Covidien Endo GIA™/Signia™ with Tri-Staple™ reloads Technology (Covidien/Medtronic, Minneapolis, MN, USA). During this study period, staple line buttress materials were introduced and incorporated. Two main types of buttressing materials were biocompatible glycolide copolymer buttress (Seamguard®), W.L. Gore & Associates, Inc., Flagstaff, AZ, and polyglycolic acid used in the Tri-Staple™ 2.0, Covidien/Medtronic, Minneapolis, MN, USA.

Water soluble contrast swallow was performed on day 1 post-operative to assess the sleeve anatomy, liquid transit and exclude early postoperative leaks. All patients underwent a modified diet protocol of gradual transition from liquid to semi-solid diet over 6 weeks post-operatively. Proton pump inhibitor was prescribed for 4 weeks post-operative and continued for 4 to 12 weeks. A normal diet was instituted after 6 weeks.

In a two-stage revision, any non-infected or eroded gastric band system was removed in the initial stage, with unfolding of the stomach and removal of sutures and band capsule where feasible. In contrast, patients with band infection or erosion will have the band system removed in the first stage without removal of the gastric plication sutures or capsule excision. This was aimed to minimise unnecessary dissection that may further disrupt the gastric wall defect and impede healing. A subsequent contrast swallow study was performed to confirm healing. Following an interval of at least 3 months for recovery, a RLSG was performed using the technique described above.

### Follow-up

Patients were routinely followed up by the operating surgeon, bariatric physicians and dietitians. Our protocol aimed to see patients at 4 weeks, 4 months and 12 months during their first post-operative year. Follow-up then was aimed to be yearly indefinitely. Patients were educated with dietary counselling at each visit. This involved discussions with the patient about chewing well (up to 20 times), avoiding eating to the point of excessive fullness, portion size and texture selection [19]. It was ensured that all staff seeing patients endeavoured to provide that information, which was reinforced annually. Additionally, from 2016, a policy of routine surveillance gastroscopies was adopted.

### Outcome Measures

The following outcome measures were collated:

1. Pre-operative baseline demographics
  - Weight and BMI
  - Previous band surgeries
  - Duration from LAGB to LSG
  - Indication for LAGB revision
  - One stage vs two stage procedure
2. Peri-operative outcomes and complications
3. Hospital resource use:
  - Length of postoperative hospital stay
  - Unplanned re-admissions
  - Unplanned return to theatre
  - Unplanned ICU admission
  - Re-operation rates
4. Weight-loss outcomes
5. Adverse symptoms
6. Patient satisfaction with surgery
7. Quality of life

Excess weight was defined as the difference between the initial weight (weight at operation, kg) and ideal weight (kg) (BMI 25 × height^2^).

Patients that had not been seen in 2.5 years or more were classified as lost to follow-up. We selected the recorded annual weight of the patients and rounded it to the closest year of follow-up for our results.

### Questionnaire Collection

A previously described self-reported questionnaire was modified to assess LSG outcomes [20]. The questionnaire used standardised symptom score scales to measure satiety and frequency of adverse symptoms (dysphagia, heartburn and regurgitation). Quality of life was scored using the Rand SF-36 Health Survey [21]. Satisfaction with surgery was measured on a scale of 0 (unsatisfied) to 10 (very satisfied).

### Statistical Analysis

Continuous parametric variables were presented as means and standard deviation, while non-parametric data were presented as median and interquartile range (IQR). The Mann–Whitney *U* test was used to compare non-parametric continuous variables, while categorical data were analysed using the chi-square and Fisher’s exact tests and presented as percentages. A two-sided *p*-value of 0.05 was considered statistically significant. Univariate binary logistic regression was performed to identify the relationship between each variable and the outcome. Any confounding variables were adjusted for in the multivariate binary logistic regression model with stepwise backward (Wald). To assess for colinearity in the regression model, we used the variance inflation factor (VIF) that identified any correlation between the independent variables and the strength of that correlation. Omnibus tests of model coefficients were used to determine the overall model fit and its statistical significance. Nagelkerke R2 method was used to determine the variation in the model. Additionally, survival curves were obtained with the Kaplan–Meier estimate. Statistical analysis was performed using SPSS version 28 (SPSS Inc., Chicago, IL, USA) and GraphPad Prism version 9.1.2 (GraphPad Software, San Diego, CA, USA).

## Results

### Baseline Characteristics

A total of 1800 patients were included in the study. The RLSG group consisted of 600 patients, and the control group consisted of 1200 PLSG patients. This data is summarised in Table 2.

**Table 2:** Baseline patient demographics

Variable	Revision LSG	Controls	*p*-value
Number	600	1200	
Age at operation (years)	45 ± 19.4	44.6 ± 16.6	0.561
Female gender (%)	87	78.8	0.132
Baseline weight (kg)	119.7 ± 26.2	120.6 ± 26.5	0.961
Baseline BMI (kg/m^2^)	43.8 ± 8.9	42.3 ± 8.1	0.934
Excess weight (kg)	51. 3 ± 24	53.4 ± 23.5	0.473
BMI ≥ 50, *N* (%)	123 (21%)	276 (23%)	0.865
Number of band revisions *N* (%)			
*N* = 0	431 (72%)		
*N* = 1	129 (22%)		
*N* = 2	42 (7%)		
*N* = 3	1 (0.2%)		
Band to sleeve conversion			
One-stage	122 (20.3%)		
Two-stage	472 (80%)		
Indication for revision surgery			
Eroded band	22 (3%)		
Device failure- Leaking band	12 (2%)		
Transhiatal gastric enlargement	116 (19.3%)		
Transhiatal oesophageal enlargement	201 (33.5%)		
Pan oesophageal dilatation	34 (5.7%)		
Anatomically normal (weight loss failure/ patient choice)	218 (36.3%)		

Student’s *t* test for continuous data and the chi-squared test for categorical data, unless otherwise specified

A subgroup analysis was conducted based on the classification of band complication and indication for RLSG.

1. Eroded bands
2. Focal luminal dilatation:
  - Gastric enlargements
  - Oesophageal enlargements
3. Pan oesophageal dilatation and severe oesophageal dysmotility
4. Oesophageal dysfunction (dysmotility/hypersensitivity)—with anatomically normally sited gastric band

Band removal was primarily due to weight loss failure (36.3%). The median time between LAGB placement and LSG was 91 (IQR 68) months, with 80% (*n* = 472) conducted as two-stage conversion procedures; 8/600 (1.3%) conversions were open procedures.

The outcomes between one-stage and two-stage conversions of LAGB to LSG are shown in supplementary Table 1.

### Peri-operative Outcomes

#### Revision LSG Group

**Fig. 3:**
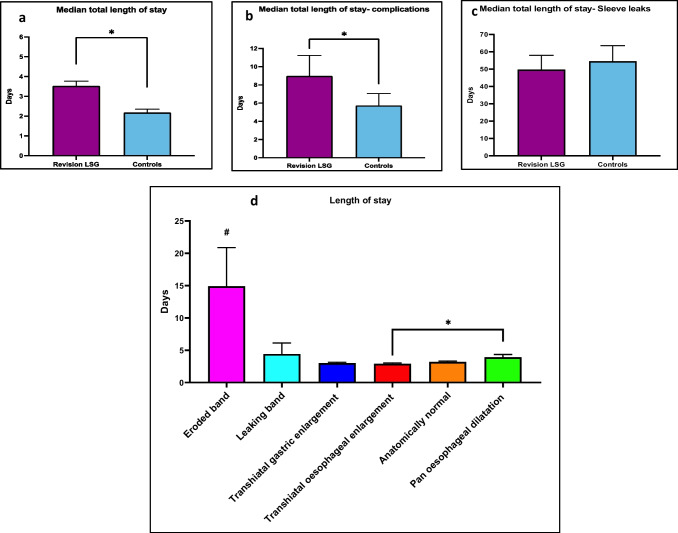
Length of stay in revisional sleeve gastrectomy. **a** Median total length of stay for admissions related to revisional LSG and controls. **b** Median total length of stay for complications related to revisional LSG and controls. **c** Median total length of stay for sleeve leaks between revisional LSG and controls. **d** Median total length of stay for admissions related to indications for band revision; the symbol # indicates significance between eroded band and all groups. Revisional LSG (*n* = 600) and controls (*n* = 1200)

The median length of stay was significantly longer in RLSG compared with the controls 3 (IQR 2) days vs 2 (IQR 2) days, *p*-value 0.001 (Fig. 3a). Furthermore, RLSG patients with post-operative complications had a longer length of stay 4 (IQR 4) days vs 3 (IQR 2) days, *p*-value 0.001 (Fig. 3b).

Thirty-day complications, re-operations and re-admissions rates are presented in Table 3. The RLSG group had overall higher complications (4.8% vs 2.0% RR 2.4, *p*-value < 0.0001) and re-admissions (4.3% vs 2.4% RR 1.8, *p*-value 0.012).

**Table 3:** Comparison of outcomes between revision laparoscopic sleeve gastrectomy and controls

Parameter	Revision LSG	Controls	*p*-value^
*n*	600	1200	
30-day peri-op outcomes			
30-day mortality	1 (0.2%)	0	0.333
30-day unplanned re-admission	26 (4.3%)	29 (2.4%)	**0.012**
30-day return to theatre	9 (1.5%)	11 (0.9%)	0.266
30-day unplanned ICU admission	2 (0.3%)	3 (0.3%)	0.999
Sleeve complications			
Leak *Clavien Dindo classification*	15 (2.5%)	11 (0.9%)	**0.011**
Grade II	2 (0.3%)	5 (0.4%)	0.999
Grade IIIa	2 (0.3%)	3 (0.2%)	0.999
Grade IIIb	5 (0.8%)	2 (0.1%)	**0.044**
Grade IVa	5 (0.8%)	1 (0.08%)	0.110
Grade IVb	1 (0.2%)	1 (0.08%)	0.097
*Johari Radiological classification*			
1a	0	1 (0.08%)	0.999
1b	1 (0.2%)	1 (0.08%)	0.097
2a	3 (0.5%)	3 (0.2%)	0.999
2b	1 (0.2%)	3 (0.2%)	0.999
3a	5 (0.8%)	1 (0.08%)	**0.031**
3b	5 (0.8%)	2 (0.1%)	**0.044**
4	0	0	
Stenosis	0	1 (0.08%)	0.999
Fistula	2 (0.3%)	1 (0.08%)	0.097
Thrombosis	3 (0.5%)	5 (0.4%)	0.725
Wound problems	4 (0.7%)	3 (0.2%)	0.108
Major bleed*	2 (0.3%)	4 (0.3%)	0.688
Sepsis	3 (0.5%)	1 (0.08%)	0.090
30–90 days post-op outcomes			
30–90 day mortality	0	0	
30–90 day unplanned re-admission	22 (3.7%)	3 (0.2%)	** < 0.0001**
30–90 day return to theatre	2 (0.3%)	1 (0.08%)	0.091
30–90 day unplanned ICU admission	1 (0.2%)	0	0.999
Sleeve complications			
Leak	2 (0.3%)	3 (0.2%)	0.721
*Clavien Dindo classification*			
Grade II	0	0	
Grade IIIa	0	0	
Grade IIIb	1 (0.2%)	0	0.999
Grade IVa	1 (0.2%)	0	0.999
Grade IVb	0	0	
*Johari Radiological classification*			
1a	0	1 (0.08%)	0.999
1b	0	1 (0.08%)	0.999
2a	1 (0.2%)	0	0.999
2b	1 (0.2%)	0	0.999
3a	0	0	
3b	0	0	
4	0	0	
Stenosis	0	0	
Fistula	1 (0.2%)	1 (0.08%)	0.097
Thrombosis	0	1 (0.08%)	0.999
Wound problems	1 (0.2%)	0	0.999
Major bleed*	0	0	
Sepsis	1 (0.2%)	0	0.999
> 90 days post-op outcomes			
> 90 day mortality	0	0	
> 90 day unplanned re-admission	4 (0.7%)	5 (0.4%)	0.509
> 90 day return to theatre	1 (0.2%)	0	0.999
> 90 day unplanned ICU admission	0	0	
Sleeve complications			
Leak	2 (0.3%)	1 (0.08%)	0.621
*Clavien Dindo classification*			
Grade II	0	2 (0.1%)	0.999
Grade IIIa	1 (0.2%)	0	0.999
Grade IIIb	0	0	
Grade IVa	1 (0.2%)	1 (0.08%)	0.721
Grade IVb	0	0	
*Johari Radiological classification*			
1a	0	0	
1b	0	0	
2a	0	0	
2b	0	0	
3a	0	0	
3b	0	0	
4	2 (0.3%)	1 (0.08%)	0.621
Stenosis	1 (0.2%)	0	0.999
Fistula	0	0	
Thrombosis	0	0	
Wound problems	1 (0.2%)	0	0.999
Major bleed*	0	0	
Sepsis	2 (0.3%)	1 (0.08%)	0.621

^*^Bleeding requiring re-operation or transfusion

^*p*-value calculated using chi-square test

The bolded values highlight the significant p-values

Compared to the control group, overall RLSG had more frequent staple line leaks (0.9% vs 2.5% RR 1.8, *p*-value 0.011). On average, the RLSG patients were diagnosed 9.5 ± 6.9 days post-surgery. Leaks were diagnosed using water-based soluble contrast (Gastrografin) swallows for 3/15 patients, with the remaining patients diagnosed using Computer Tomography (CT) scans with on-table contrast. The leak site was observed at the proximal compartment of the sleeve in all patients. The median length of stay for RLSG patients experiencing sleeve leaks was 22 (8–111) days. Thirty-three percent of patients (*n* = 5) were conservatively managed using antibiotics. Multiple interventions were performed in 6/15 RLSG patients. Endoscopic, radiological and surgical management for the more severe leaks are as follows: stents (*n* = 4), glue (*n* = 2), endoscopic vacuum therapy (*n* = 3), radiological drainage (*n* = 5) and surgical procedures (*n* = 6).

#### Indications of LAGB Conversion (Eroded Bands and Pan Oesophageal Dilatations)

Overall, the length of stay was significantly different among the groups (*p*-value < 0.001) (Fig. 3d). A total of 22 RLSG patients (3.7%) had prior eroded bands. The time between band removal for erosion and RLSG was a median of 1.2 years (0.6 to 12.7 years). Overall complication rates (65%, RR 31.8, *p*-value < 0.0001) and readmission rates (55%, RR 22.5, *p*-value < 0.0001) were markedly high in the eroded band patient group. They also had a significantly longer length of stay than the other groups (14.0 ± 26.8 days). Fifty percent of these patients experienced sleeve leaks (*n* = 11). Additionally, the time between band removal for erosion and RSLG was further categorised into three groups: early (< 6 months), intermediate (6–12 months) and late (> 12 months). Those in the intermediate (100%, *n* = 6) to late (80%, *n* = 4) groups had a higher percentage of patients experiencing leaks compared to those in the early group (25%, *n* = 1), *p*-value < 0.0001. Other complications included major bleeds (*n* = 2), strictures (*n* = 1), dysphagia (*n* = 4), nausea (*n* = 2) and vomiting (*n* = 2).

Those who were converted due to pan oesophageal dilatations had higher complication (15% RR 6.9, *p*-value < 0.0001) and readmission rates (12% RR 4.9, *p*-value < 0.0001) when compared with the other CORE physiological groupings. The following complications were noted: sleeve leak (*n* = 4), stricture (*n* = 2), tachycardia (*n* = 1), fever (*n* = 1), bleed (*n* = 1), infected seroma (*n* = 1) and wound dehiscence (*n* = 1) leading to a significantly longer length of hospital stay (3.9 ± 2.4 vs 2.9 ± 2.1 days, *p*-value 0.025).

### Predictors of Complications

Using multivariate regression analysis, only eroded bands, number of band revisions and baseline weight were independent predictors of complications after RLSG. The adjusted odds of having a complication among eroded band patients are 6.9-fold higher, and the odds are significant (*p*-value 0.001, 95% CI: 2.5–18.9).

Furthermore, the odds of complications among those who have had two revision band procedures are 2.9-fold higher (*p*-value 0.049, 95% CI: 1.0–8.6).

With every unit (kg) increment in baseline weight, the odds of having a complication increase by 8.8% (*p*-value 0.043, CI: 0.98–1).

This model was found to be statistically significant (chi-square 19.4, *p*-value 0.013), whereby the model explained 69% (Nagelkerke *R*-square) of the variance in complication outcomes following RLSG. Age, gender, baseline BMI, excess weight and stage of revision (one vs two) were adjusted for in the model.

### Mortality

Two mortalities occurred in the RLSG group (0.3%). One mortality was due to a sleeve leak (2 days post-discharge). The second was 12 months post-op and not deemed related to surgery. There were no mortalities in the control group.

### Follow-up

Maintenance of overall follow-up was achieved for 87% of RLSG patients and 89.3% of controls, respectively. Overall lost to follow-up for RLSG patients (15%) and 216 controls (18%), *p*-value 0.386. Their weight loss and revisional surgery data have been included until their last visit.

### Weight-Loss Outcomes

#### Revision LSG Group

**Fig. 4:**
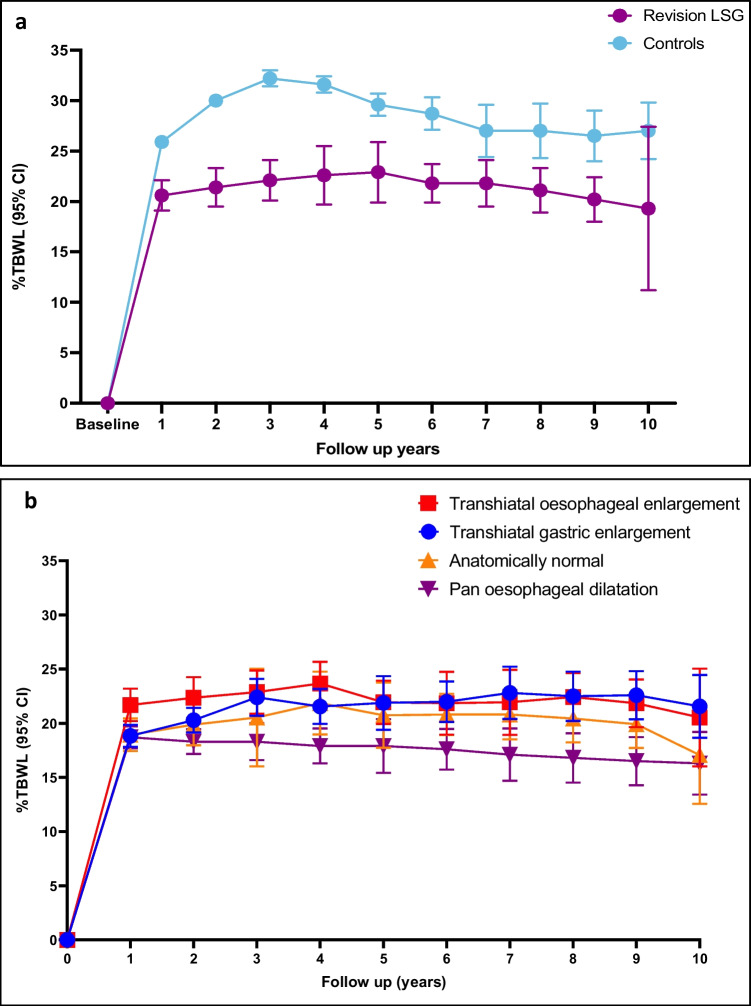
Percentage total weight loss with 95% CIs for the 10 year follow up period. **a** %TWL comparing revisional LSG (*n* = 600) and controls (*n* = 1200). **b** %TWL comparing indications for band revision

Weight loss over the 10-year follow-up is shown as % total weight loss (%TWL) in Fig. 4. The controls experienced more significant weight loss over the 10 years compared with the RLSG procedure (*p*-value < 0.0001). Maximal weight loss in RLSG had reached at 5 years, 22.9% TWL (*n* = 68) and remained relatively stable from 5 to 10 years with a mean of 20.9% TBWL.

#### Indications of LAGB Conversion

Sub-analysis for weight loss outcomes based on RLSG indications showed those who had transhiatal enlargements had a maximal weight loss of 23.7% TWL after 4 years. Overall, the pan oesophageal dilatation group reached maximal weight loss of 18.7% TBWL at 1 year, reduced over the years and reached 16.3% TWL at the 10-year mark. Overall, this group of patients had a significantly lower weight loss than the other groups based on indication for surgery (*p*-value 0.035).

### Revisional Surgery

**Fig. 5:**
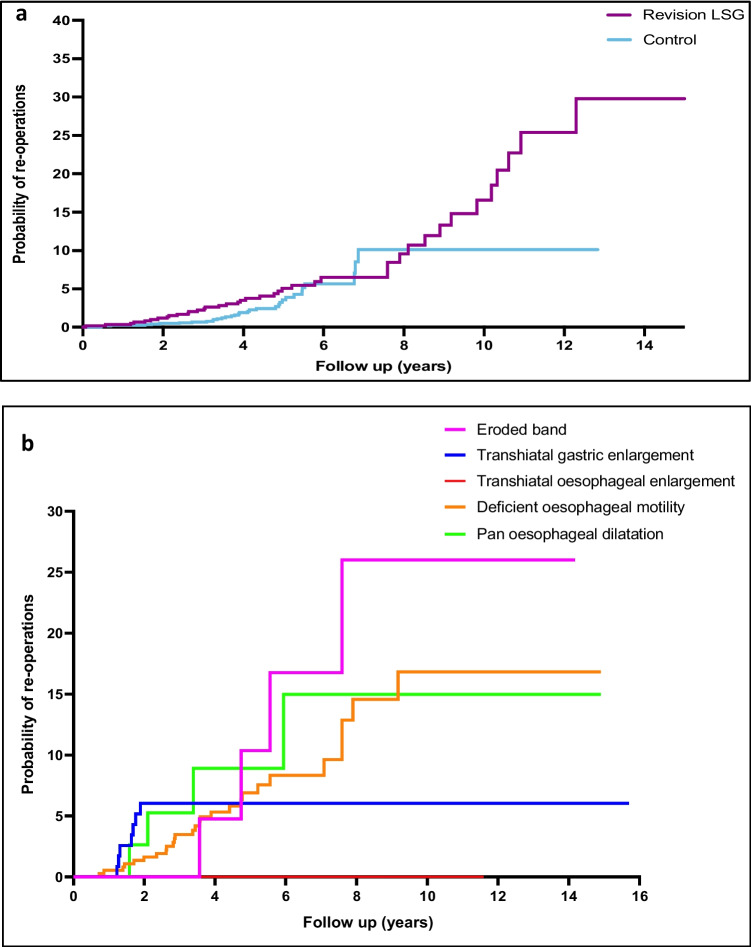
Kaplan–Meier curve of the probability for re-operations as a function of time (years) in sleeve gastrectomy. **a** Probability for re-operations comparing revisional LSG (*n* = 600) and controls (*n* = 1200). **b** Probability for re-operations comparing indications for band revision

RLSG (7.3%) had a higher rate of subsequent revisional surgery compared to the controls (3.2%) (RR 4.8, *p*-value < 0.0001). The revisional surgery rates for both groups of patients are shown in Table 4. The indications were classified as operative complications, adverse symptoms (intolerance of oral intake, reflux) and weight regain. Reflux and weight regain were the most common indication for revision in both groups. Roux-en-Y gastric bypass (RYGB) was the most popular revision procedure in both groups. However, the rate of conversion to RYGB was higher in the RLSG group (3.3%) compared to the controls (1.2%) (*p*-value < 0.0001). The probability of having a re-operation at 5 years was 5.2% for RLSG and 3.3% for controls. Notably, Kaplan–Meier analysis estimated a long-term re-operation rate of 30% over 14 years in the RLSG group, compared to 10% in the controls, *p*-value 0.031 (Fig. 5a).

**Table 4:** Long-term readmissions and re-operations in revision LSG patients

Indication for readmission	Revision LSG, *n* (%)	Controls*, n* (%)	*p*-value^
*n*	600	1200	
Cholecystectomy	3 (0.5)	23 (1.9)	0.317
Intolerance of oral intake Conservatively managed Sleeve stricture requiring dilatation	3 (0.5) 3 (0.5) 1 (0.2)	7 (0.6) 1 (0.1) 6 (0.5)	0.854 0.212 0.231
Incisional hernia repair	4 (0.7)	4 (0.3)	0.971
Abdominal pain, no cause identified	2 (0.3)	5 (0.4)	0.384
Nutritional deficiency	3 (0.5)	3 (0.3)	0.421
Revisional bariatric operation (elective)			
Total	50 (8.3)	38 (3.2)	** < 0.0001**
Procedure breakdown			
Roux-en-Y bypass	32 (5.3)	14 (1.2)	** < 0.0001**
One anastomosis bypass	6 (1.0)	16 (1.3)	0.603
Subtotal gastrectomy Roux-en-Y	4 (0.5)	2 (0.2)	0.323
Biliopancreatic diversion	8 (1.3)	2 (0.2)	** < 0.0001**
Indication			
Adverse symptoms			
Intolerance of oral intake	2 (0.3)	2 (0.2)	0.276
Reflux	20 (3.3)	9 (0.8)	** < 0.0001**
Weight gain	30 (5.0)	20 (1.7)	** < 0.0001**

^*p*-value calculated using chi-square test

The bolded values highlight the significant p-values

#### Re-operation Based on Indications for LAGB Conversion

There was a high rate of overall long-term re-operations in those who had RLSG following eroded bands (18%), anatomically normal (12.4%) and pan oesophageal dilatation (11.8%) in comparison to the remaining CORE classification groups, *p*-value 0.003. The majority of these patients were converted to the RYGB procedure.

### Patient-Reported Experience

#### Adverse Symptoms

**Fig. 6:**
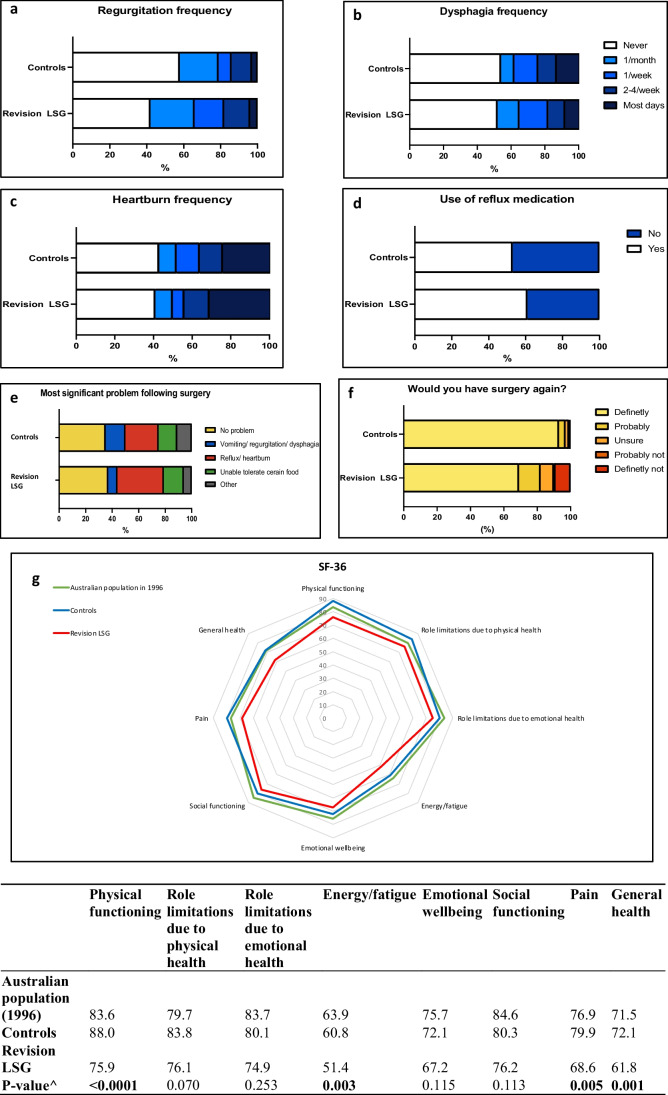
Patient-reported outcome measures. Responses to **a** ‘regurgitation frequency’, **b** ‘dysphagia frequency’, **c** ‘heartburn frequency’, **d** ‘use of reflux medication’, **e** ‘most significant problem following surgery’, **f** ‘would you have the surgery again?’ and **g** SF-36 quality of life, ^*p*-value calculated using one-way ANOVA

Adverse symptoms experienced by RLSG and controls are shown in Fig. 6. Regurgitation was the most frequently experienced symptom in RLSG (58%) compared to controls (42%), *p*-value 0.034. Bothersome regurgitation was substantially elevated in RLSG patients, with 42.5% of patients moderately or severely bothered vs 12% of controls.

Food tolerance differed between the two groups. In total, 65.4% of RLSG patients avoided moderate or many foods compared with 21.3% of PLSG patients (*p*-value < 0.0001). However, the composite dysphagia scores were high and similar in both groups (29 ± 12.3 vs 32.2 ± 12.1, *p*-value 0.126), with 0 representing frequent dysphagia and 45 representing no dysphagia).

Composite reflux scores were also low and similar in both groups (13.0 ± 15.2 vs 11.1 ± 13.9, *p*-value 0.210), with 0 representing no reflux and 72 representing maximum reflux.

### Patient Perspective on Surgery

Patients were highly satisfied with the procedure (7.9 ± 2.8 in RLSG vs 9.0 ± 1.9 in controls, *p*-value 0.005, score out of 10, 0 being definitely no and 10 being definitely yes). Most patients would undergo surgery again if given the option in both groups. However, this percentage was higher in the control group (93% vs 69%, *p*-value < 0.0001). The most bothersome symptom or problem following the surgery was reflux/heartburn. This data is shown in Fig. 6e–f.

### Post-operative Satiety

Pre-meal satiety scores were similarly reported in both groups in the morning 5.2 ± 2.6 vs 4.9 ± 2.1 (*p*-value 0.417), at lunch 4.5 ± 2.8 vs 4.3 ± 1.8 (*p*-value 0.667) and dinner 3.4 ± 2.3 vs 3.9 ± 1.7 (*p*-value 0.421) (score of out 10, 0 being extremely hungry and 10 being extremely full).

### Quality of Life

Quality of life was assessed across eight domains using the Rand SF-36 assessment. RLSG quality of life was significantly less than the controls and community normal scores. Comparisons between the two sleeve groups demonstrated significant differences in physical function, energy/fatigue, pain and general health scores. These data are shown in Fig. 6g.

## Discussion

We have completed a detailed evaluation of the long-term outcomes of sleeve gastrectomy after laparoscopic adjustable gastric banding in a large cohort of 600 RLSG patients with a matched control group of PLSG patients. RLSG patients achieved and sustained substantial long-term weight (20.9% TWL at 10 years) although this was significantly lower than the control group (27% TWL). Whilst 30-day re-admission, complications and mortality rates were low after RLSG; they were significantly higher than the control group.

A key finding was that RLSG appeared to have a markedly increased probability of further re-operation over time with a projected rate of 30% at 14 years, whereas controls had a revision rate of 10%. Overall satisfaction with RLSG was moderate and much significantly lower than with controls. RLSG patients also reported more bothersome regurgitation (58%), poorer food tolerance and a lower quality of life than the controls.

Eroded LAGB, multiple prior band operations, oesophageal dilatation and elevated baseline weight significantly increased the risk of peri-operative complications, poor weight loss and long-term re-operation in the RLSG group. These factors should be considered when discussing conversion of LAGB to LSG.

The most significant complication in the RLSG group was sleeve leaks with an incidence of 2.5%, compared to 0.9% in the control group. When compared to a study conducted by Spaniolas et al. (2017) in a large cohort of 3364 LAGB to LSG patients, a significantly lower sleeve leak rate of 0.8% was reported [22]. The difference found in our patient cohort could be due to a higher proportion of low grade leaks as the incidence of severe leaks and those requiring interventions was low. It would be ideal to compare series using an objective classification system. The Johari grading system is highly sensitive in identifying leaks and that data has shown to closely correlate with outcomes [23]. This grading system provides a valuable tool for comparing the true incidence of sleeve leaks and their resolution and overcomes the confounder of variable definitions and data collections used across different centres.

Importantly, 50% of patients who had an eroded gastric band prior to RLSG experienced a sleeve leak. The higher leak rates observed in RLSG could potentially be attributable to a lower threshold for disruption of the staple line from isobaric pressurisation due to the thickened tissue around the cardia and fundus, the formation of a fibrotic capsule at the site of the band and lastly, interrupted blood supply with frayed, thinned luminal wall [24, 25].

We observed staple line leaks when greater than 1 year had elapsed between removal and RLSG. It appears to us that most likely the pathology of the eroded band permanently disrupts the transmural vascularity of the stomach, and this would not be expected to substantially recover over time. On average patients had gained 12 kg since their band removal surgery, which may also have contributed to an increased risk of leaks.

Single versus two-stage conversion of LAGB to SG remains controversial. A large proportion (80%) of patients in our study underwent a two-stage LAGB to LSG procedure. This is reflective of our local practices and that many patients had undergone complex previous gastric band surgery with prior complications and revisions. There was an evolution towards single-stage surgery; however, some surgeons remain wary of that approach given there is conflicting literature.

Our structured algorithm with pre-operative and intra-operative criteria appears objective, and successful facilitated equivalent outcomes to two-stage procedures were observed. We suspect that different indications and approaches result in differing outcomes. Our study included 122 subjects undergoing single-stage conversion with similar results to other series [26].

A unique feature of this study was our physiological and anatomical stratification of the indication for LAGB conversion to LSG according to the previously described CORE classification of LAGB complications, using barium swallow, gastroscopy and selective application of high-resolution manometry. There was a high rate of overall long-term re-operations in those who had eroded bands (18%), deficient motility (12.4%) and pan oesophageal dilatation (11.8%), *p*-value 0.003. Intact peristalsis is required to drive transit through the sleeve and is deemed intrinsic to normal sleeve functioning [27]. These conditions impede motility and thus normal sleeve function and are most likely why poorer outcomes were observed [28].

Most studies that have examined conversion of LAGB to LSG have reported weight loss, however, without a comparison group [29–31]. Angelis et al. were one of the few studies comparing 44 RLSG patients to 56 PLSGs, showing a mean TWL of 26 ± 12% at 8 years in RLSG. This differed significantly from their PLSG group [12]. Our results were also comparable at the 8-year mark. Several other series that compared RLSG to PLSG also showed significant weight loss differences between the two groups [13, 32].

The high incidence of reflux following sleeve gastrectomy is an ongoing challenge for both patients and surgeons. Arman et al. (2016) and Felsenreich et al. (2016) reported that 31.7% and 36% of patients underwent reoperation for weight regain or GERD [33, 34]. This significantly differed from the 3.3% (reflux) and 5% (weight regain) reported by our cohort.

Key strengths of this study include the large numbers of the first 600 patients undergoing RLSG that have been well matched for baseline demographics and a control group. Analysing a broad range of outcomes has provided a comprehensive overview of clinical, hospital resource use and patient-reported data.

We have verified and increased our data’s completeness by crosslinking with prospectively maintained databases, including locally held databases and the national registry data providing high long-term follow-up rates. This allows us to be confident of the robustness of the data. In addition, we have used the large numbers to perform sub-group analysis based on the indications for surgery and provide additional statistical projections of revisional surgery over the longer term.

There are several limitations in this study. Whilst we were able to attain insights into the differences in outcomes, it was statistically underpowered to evaluate and compare adequately across different outcome measures. Our study, however, is representative of the overall outcomes of RLSG, as this was the main procedure we have offered to this patient cohort. This may be an area to address in the future, and comparative studies, reporting on a comprehensive range of outcomes in the long term, are required. This study was underpowered to explore sub-group analysis of all secondary outcome measures, and that is not surprising as our primary aim was to evaluate long-term weight loss, peri-operative risk and re-operation rates. This does provide the opportunity for future more targeted studies.

## Conclusion

LSG performed as a revisional procedure provides effective long-term weight loss, although peri-operative complications are significantly elevated compared to primary procedures. The requirement for longer-term re-operations appears elevated at an estimated 30% compared to only 10% with primary procedures. Patient satisfaction with surgery is moderate following RLSG, although quality of life is less than with PLSG and adverse gastrointestinal symptoms are more prevalent, potentially explaining the higher re-operation rates.

Stratification by anatomical and physiological indication for conversion from LAGB to LSG demonstrates significant differences in outcomes and is a valuable adjunct. Four situations following LAGB predict substantially worse outcomes: eroded gastric bands, multiple prior bands, oesophageal dysfunction and increased body weight. Eroded bands demonstrate a substantially increased risk of leaks that should preclude LSG. These data provide a framework for understanding the expected long-term outcomes and specific criteria for delineating situations that are at much higher risk of worse overall outcomes.

### Supplementary Information

Below is the link to the electronic supplementary material.

Supplementary file1 (DOCX 18345 KB)
