# Supplementary material for: Long-term Outcomes of Laparoscopic Sleeve Gastrectomy as a Revisional Procedure Following Adjustable Gastric Banding: Variations in Outcomes Based on Indication
Source: Obes Surg. 2023 Oct 17;33(12):3722–39. doi: 10.1007/s11695-023-06886-8 (PMC10687173; doi:10.1007/s11695-023-06886-8)
Supplement: Supplementary file 1 — Supplementary file1 (DOCX 18345 KB) [file 11695_2023_6886_MOESM1_ESM.docx]

**Supplementary tables**

**Table 1: Baseline patient demographics, post-operative and weight loss outcomes of one-stage vs two-stage sleeve gastrectomy conversions**

| **Variable** | **One-stage** | **Two-stage** | **P-value** |
| --- | --- | --- | --- |
| Number | 122 | 472 |  |
| Age at operation (years) | 45.1 ± 9.4 | 45.1 ± 9.4 | 0.983 |
| Female gender (%) | 108 (88.5) | 401 (85) | 0.456 |
| Baseline weight (kg) | 120.3 ± 25.7 | 119.5 ± 26.4 | 0.750 |
| Baseline BMI (kg/m^2^) | 43.5 ± 8.1 | 43.8 ± 9 | 0.705 |
| Excess weight (Kg) | 51. 2 ± 23.4 | 51.3 ± 24.4 | 0.974 |
| *Outcomes* |  |  |  |
| Length of stay, days | 3.5 ± 2.8 | 3.6 ± 6.5 | 0.666 |
| 30-day mortality | 1 (0.8%) | 1 (0.2%) | 0.316 |
| 30-day unplanned re-admission | 6 (4.9%) | 21 (4.4%) | 0.733 |
| 30-day return to theatre | 4 (3.3%) | 6 (1.3%) | 0.312 |
| 30-day unplanned ICU admission | 0 | 2 (0.4%) | 0.843 |
| 30-day sleeve leaks | 8 (6.6%) | 15 (3.2%) | 0.189 |
| >30-day mortality | 0 | 0 | - |
| >30-day unplanned re-admission | 1 (1%) | 7 (5%) | 0.033 |
| >30-day return to theatre | 0 | 1 (0.2%) | 0.985 |
| >30-day unplanned ICU admission | 0 | 0 | - |
| >30-day sleeve leaks | 1 | 0 | 0.985 |
| *Weight loss outcomes* |  |  |  |
| %TWL 1 year, mean (CI) | 18.9 (1.6) | 22.7 (1.1) | 0.050 |
| %TWL 2 year, mean (CI) | 19.8 (1.9) | 20.7 (0.8) | 0.225 |
| %TWL 3 year, mean (CI) | 19.8 (1.8) | 17.6 (3.6) | 0.609 |
| %TWL 4 year, mean (CI) | 19 (1.7) | 17.9 (3.2) | 0.282 |
| %TWL 5 year, mean (CI) | 17.5 (1.3) | 19.6 (0.6) | 0.181 |
| %TWL 10 year, mean (CI) | 17.9 (2.2) | 19.1 (1.5) | 0.789 |

**Figure 1**

**Figure 1: Intraoperative images of sleeve gastrectomy a** Image showing gastric surface contour (Grade 2) and surface debris (Grade 2) at the proximal component of the stomach from previous LAGB. **b** Image showing a gastro-gastric tunnel where LAGB used to be. **c** Image showing a significantly distorted shape of gastric wall (Shape 3) with a large proximal gastric pouch with a gastric band insitu. **d** Image showing an unbuckled gastric band with an intact gastro-gastric tunnel and a distorted shape of gastric wall (Grade 3). **e** Image showing extensive infolding of the gastric surface contour (Grade 3). **f** Image showing a poor quality of gastric wall (Grade 3) with excessive intramural scarring and fibrosis. **g** and **i** Image showing stomach a relatively normal configuration of the stomach suitable for single stage conversion.
